# One high quality genome and two transcriptome datasets for new species of *Mantamonas*, a deep-branching eukaryote clade

**DOI:** 10.1038/s41597-023-02488-2

**Published:** 2023-09-09

**Authors:** Jazmin Blaz, Luis Javier Galindo, Aaron A. Heiss, Harpreet Kaur, Guifré Torruella, Ashley Yang, L. Alexa Thompson, Alexander Filbert, Sally Warring, Apurva Narechania, Takashi Shiratori, Ken-ichiro Ishida, Joel B. Dacks, Purificación López-García, David Moreira, Eunsoo Kim, Laura Eme

**Affiliations:** 1 Unité d’Ecologie Systématique et Evolution, CNRS, Université Paris-Saclay, AgroParisTech, Gif-sur-Yvette, France; 2 Department of Biology, University of Oxford, Oxford, United Kingdom; 3 Institute of Life and Environmental Sciences, University of Tsukuba, Tsukuba, Japan; 4 Division of Invertebrate Zoology, American Museum of Natural History, New York, NY USA; 5 Department of Oceanography, Kyungpook National University, Daegu, South Korea; 6 Division of Infectious Disease, Department of Medicine, University of Alberta and Department of Biological Sciences, University of Alberta, Edmonton, Alberta Canada; 7 Earlham Institute, Norwich Research Park, Norwich, United Kingdom; 8 Centre for Life’s Origin and Evolution, Department of Genetics, Evolution & Environment, University College London, London, United Kingdom; 9 Division of EcoScience, Ewha Womans University, Seoul, South Korea

**Keywords:** Microbiology, Evolution

## Abstract

Mantamonads were long considered to represent an “orphan” lineage in the tree of eukaryotes, likely branching near the most frequently assumed position for the root of eukaryotes. Recent phylogenomic analyses have placed them as part of the “CRuMs” supergroup, along with collodictyonids and rigifilids. This supergroup appears to branch at the base of Amorphea, making it of special importance for understanding the deep evolutionary history of eukaryotes. However, the lack of representative species and complete genomic data associated with them has hampered the investigation of their biology and evolution. Here, we isolated and described two new species of mantamonads, *Mantamonas vickermani* sp. nov. and *Mantamonas sphyraenae* sp. nov., for each of which we generated transcriptomic sequence data, as well as a high-quality genome for the latter. The estimated size of the *M. sphyraenae* genome is 25 Mb; our de novo assembly appears to be highly contiguous and complete with 9,416 predicted protein-coding genes. This near-chromosome-scale genome assembly is the first described for the CRuMs supergroup.

## Background & Summary

Free-living heterotrophic flagellates play important roles in the nutrient cycling of marine and freshwater ecosystems. However, the extent of their genomic diversity is still dramatically uncharacterized. Amongst the lesser-known of these is *Mantamonas*, a genus of marine gliding flagellates initially described as very divergent from all other known eukaryotes^1^. Although *Mantamonas* was originally thought to be related to the poorly-known lineages Apusomonadida and Ancyromonadida, based on ribosomal RNA gene phylogenies and some of their morphological characteristics^1^, recent transcriptome-based phylogenomic analyses instead robustly placed *Mantamonas plastica* as sister to a clade comprising Collodictyonidae (also known as diphylleids) and Rigifilidae, altogether forming the “CRuMs” supergroup^2,3^. This clade presents diverse cell morphologies and branches at the base of Amorphea^2,4^ (Amoebozoa plus Obazoa, the latter including animals and fungi, among others). The genomic exploration of members of this supergroup therefore represents an important resource for uncovering the characteristics of this deep-branching clade, and may help us better understand evolutionary transitions within the eukaryotic tree of life, such as the acquisition of complex multicellularity in several lineages of the Obazoa. However, to date, only partial transcriptomic data is available for a handful of CRuMs taxa, including *M. plastica*^2,3^. Here, we isolated and described two new species of mantamonads, *Mantamonas sphyraenae* sp. nov. and *Mantamonas vickermani* sp. nov., and generated a high-quality nuclear genomic assembly for the former and transcriptomic assemblies for both species.

**Fig. 1:**
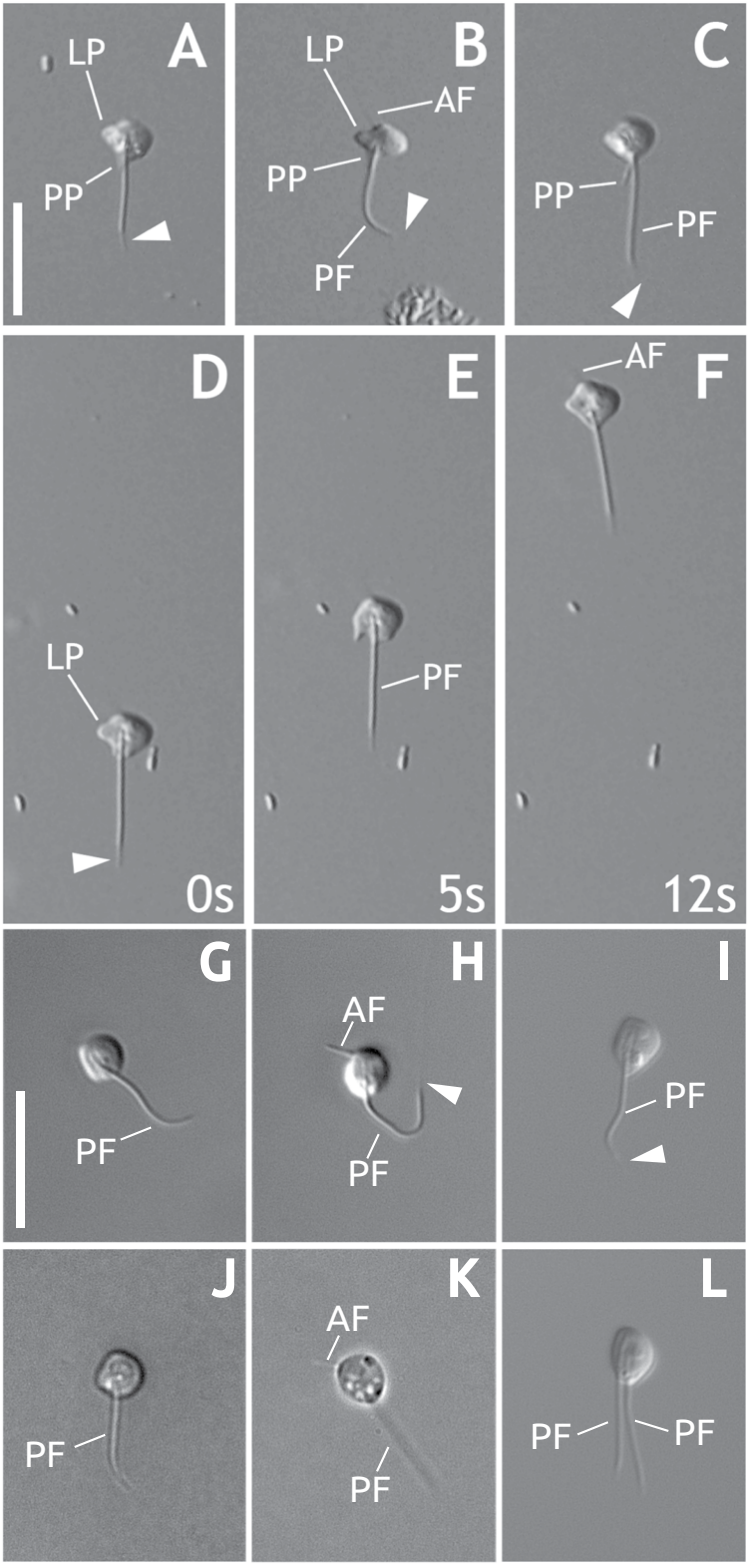
General morphology of *Mantamonas sphyraenae* sp. nov. and *Mantamonas vickermani* sp. nov. (**a**–**c**) Differential interference contrast light micrographs of living M. sphyraenae cells. Note acroneme (white arrowheads), most visible in panel (**a**) but present in all micrographs. The extremely thin anterior flagellum is visible in panel (**b**). The left projection, present in all cells, is most distinct in (**b**). A posterior protrusion is often visible, usually parallel and immediately adjacent to the posterior flagellum (**a**,**b**), but sometimes at an angle to it (**c**). (**d**–**f**) Individual M. sphyraenae cell imaged over a 12-second period; numbers in lower right indicate elapsed time in seconds. Note the plastic nature of the cell and lack of movement of the posterior flagellum except to trail behind the cell body. (**g**–**l**) Phase and differential interference contrast light micrographs of living interphase M. vickermani cells. Note contrast between thick and long posterior flagellum and thin and short anterior flagellum in (**g**,**k**). (**l**) Laterally dividing cell of M. vickermani with two posterior flagella. Scale bars: 10 μm. AF = anterior flagellum; LP = left projection; PF = posterior flagellum; PP = posterior protrusion; arrowhead = acroneme.

Overall, the cell morphology and behavior under light microscopy of these two new species (Fig. 1, Movie 1 and Movie 2) are comparable to what was reported in the original description of the genus *Mantamonas*^1^ and to our own observations of the type strain of *M. plastica*. Nonetheless, our strains appear to be slightly smaller than the 5 × 5 µm dimensions of *M. plastica*. Cells of this genus have one anterior and one posterior flagellum. They are flattened and somewhat plastic, with shapes ranging from wide, with more or less pointed lateral “wings” resembling the fins of a manta ray, to kite-shaped, to oval, to spherical. The left side of the cell body often displays a characteristic blunt projection, which we sometimes observed in our new strains, although less conspicuously (Fig. 1; see the detailed morphological description of each of the new species in *Methods* and formal species description in *Data Usage Notes*).

All previously known mantamonad strains were isolated from marine sediments^1^, which was also the case for our strain *M. vickermani* sp. nov., isolated from marine lagoon sediment. However, we isolated the other strain (*M. sphyraenae* sp. nov.) from the skin surface of a barracuda, which could suggest that either this species is epizootic (normally inhabiting the skin of the fish) or that the cells that we isolated were dislodged from their normal habitat and adhered to the fish skin by chance. Additional sampling and culturing efforts should help resolve this matter.

**Fig. 2:**
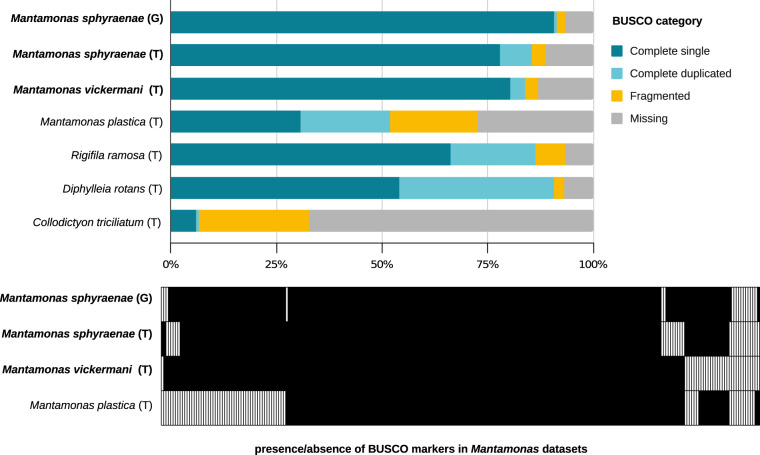
Distribution of BUSCO orthologs (eukaryota_odb10) in inferred proteomes of mantamonad assemblies of this study (in bold) in comparison with those of other members of the CRuMs supergroup. Proteomes were inferred from genome (G) and transcriptome (T) assemblies. The top panel represents the BUSCO output for each CRuMs dataset, whereas bottom panel illustrates the patterns of presence/absence of each BUSCO gene (X axis) in the four Mantamonas predicted proteomes.

**Fig. 3:**
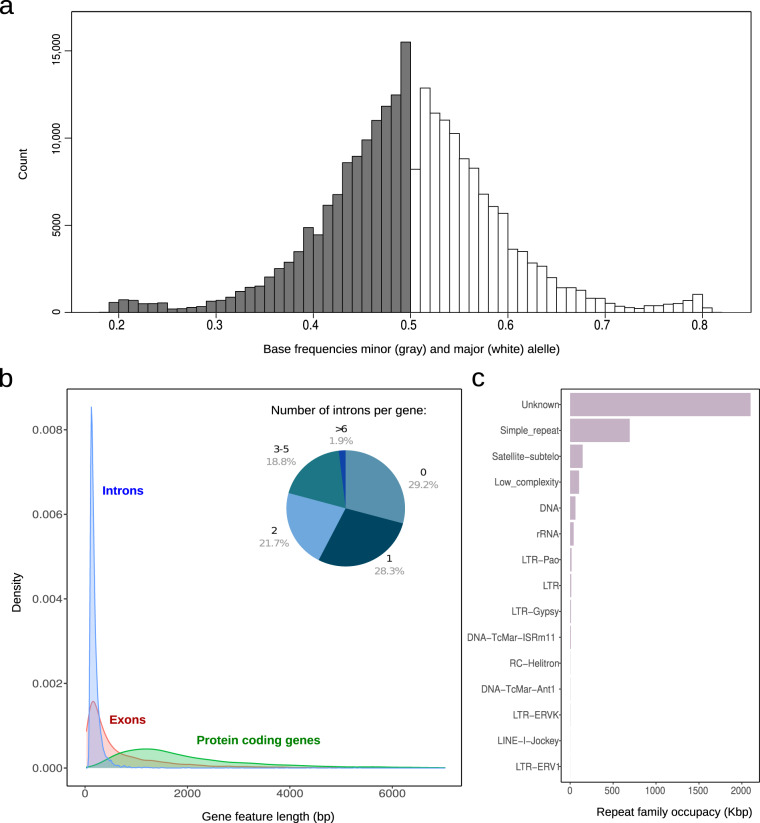
Genomic features of *Mantamonas sphyraenae* sp. nov. (**a**) Biallelic SNP frequency distribution. (**b**) Length distribution and intron frequency of protein-coding genes. (**c**) Genomic occupancy of the families of repetitive elements identified *de novo*.

The assembled nuclear genome sequence of *M. sphyraenae* is highly contiguous (Table 1). This genome sequence was generated using long (PacBio) and short (Illumina) reads (see Methods). The average sequencing coverage was 112x for PacBio and 115x for Illumina. Three different genome assembly strategies, using Canu^5^, FALCON^6^, and MaSuRCA^7^, yielded comparable results (see Methods, Table 2), with >90% representation of the 255 Benchmarking Universal Single Copy Orthologs (BUSCO^8^) of the eukaryota_odb10 dataset (Fig. 2), indicating high completeness. For downstream analyses, we opted to use the FALCON assembly because it was the most contiguous of the three, with the majority of the contigs (59 out of 78 primary contigs) bearing TTAGGG telomeric repeats at both ends. In addition, 14 of the remaining contigs had telomeric repeats at one end. While the presence of such conserved motives towards the end of the contigs suggests the complete assembly of most of the chromosomes and leads to an estimation of ~66 pairs of chromosomes in the *M. sphyraenae* nucleus, experimental evidence is needed to confirm the chromosome number in this species. Biallelic single nucleotide polymorphism (SNP) frequencies cluster around a ratio of 0.5/0.5 for each major/minor allele (Fig. 3a). This is indicative of a diploid genome, which was also supported by the statistical model of SNP frequency distribution (Table 3).

**Table 1:** Genomic and transcriptomic assemblies statistics for *Mantamonas sphyraenae* sp. nov. and *Mantamonas vickermani* sp. nov.

	*Mantamonas sphyraenae*	*Mantamonas sphyraenae*	*Mantamonas vickermani*
Assembly type	genome	transcriptome	transcriptome
Assembly length (Mb)	25.06 (31.49)	20.52	19.78
Number of contigs	78 (199)	9,255	9,796
Contig mean length (Kb)	321.30	2.218	2.019
Longest contig (Kb)	751.365	32.28	21.503
Shortest contig (Kb)	17.266	0.0202	0.0201
N50 (Kb)	375.07	3.05	2.79
L50	26	1,917	2,083
GC content	59.19	59.02	46.4
Total repeat content	12.12%	—	—

Values within parentheses correspond to primary plus associate contigs produced by FALCON.

**Table 2:** Mantamonas sphyraenae sp. nov. genome assembly statistics produced by the tested assembly strategies.

Assembly approach	Canu	Falcon	MaSuRCa
Total length (Mb)	27.35	25.06	26.11
Number of contigs	172	78	136
Mean length (bp)	159,014.56	321,299.41	191,995.24
Longest contig (bp)	732,584	751,365	1,133,621
Shortest contig (bp)	20,756	17,266	1,222
N_count	0	0	4,688
Gaps	0	0	9
N50 (bp)	303,774	375,077	386,663
N50n	32	26	24
N70 (bp)	224,361	300,753	269,297
N70n	52	41	40
N90 (bp)	50,673	226,430	146,039
N90n	95	60	65
BUSCO eukaryota odb10	C:89.1%[S:82.0%,D:7.1%], F:2.0%,M:8.9%	C:91.4%[S:90.6%,D:0.8%],F:2.0%,M:6.6%	C:89.8%[S:86.7%,D:3.1%], F:2.0%,M:8.2%

**Table 3:** nQuire Gaussian Mixture Model delta log-likelihood values for the *Mantamonas sphyraenae* genome.

Genome ploidy	Delta log-likelihood values
Diploid	10,944
Triploid	161,437
Tetraploid	104,902

The *M. sphyraenae* genome contains 9,416 predicted protein coding sequences. Genes have an average length of 2,282 bp and are mostly mono-exonic (Fig. 3b). *De novo* characterization of repetitive elements indicates that around 12% of the genome is represented by transposable elements and other repeats. While some of these were classified into different known families of DNA transposons and long terminal repeat (LTR) retroelements, the vast majority comprises unclassified types (Fig. 3c). In comparison, the transcriptome assembly of *Mantamonas sphyraenae* contains 9,256 contigs from which we predicted 8,885 non-redundant proteins and the presence of 85.5% of BUSCO eukaryota_odb10 gene set (Fig. 2). 96% of these proteins are also found in the genome-based predicted proteome, suggesting that the genome assembly represents nearly the protein repertoire represented in the transcriptome (see details in the Technical validation section, Completeness analysis).

The *de novo* assembled transcriptome of *M. vickermani* had an average sequencing coverage of 80x and led to the inference of 9,561 non-redundant proteins. As for the genome and transcriptome of *M. sphyraenae*, the proteome inferred from this transcriptome resulted in a high BUSCO score, indicating a high completeness of the predicted gene complement for this species (Fig. 2). Some BUSCO genes are consistently missing in all the four *Mantamonas* predicted proteomes, suggesting a true absence of these genes in the genus.

We inferred the phylogenetic relationships of our species within the CRuMs clade using publicly available data to reconstruct a dataset of 182 conserved protein markers and recovered the monophyly of the *Mantamonas* genus and the placement of *M. sphyraenae* as sister to a clade containing *M. vickermanii* and *M. plastica* (details in Methods, Phylogenomics analyses).

To explore the gene content diversity of our new mantamonad species, we annotated the predicted proteomes genes with EggNOG mapper^9^ and reconstructed the minimal core proteome for the genus Mantamonas and the CRuMs lineage (see details in Methods CRuMs orthologue analyses).

Finally, as an additional way of assessing the completeness of the *M. sphyraenae* and *M. vickermanii* sequence data and capturing a sense of the complexity of the cellular systems in these organisms, we interrogated the complement of one well-studied set of proteins, the membrane-trafficking system. This complex protein machinery underpins normal cellular function and is critical for feeding, cell growth, and interaction with the extracellular environment^10^. While some proteins are highly conserved across eukaryotic lineages, others have rarely been retained during evolution but were nonetheless present in the Last Eukaryotic Common Ancestor (LECA)^10^. Among them, the so-called “jotnarlogs” represent LECA proteins present in diverse extant eukaryotes but not in the major opisthokont model organisms.

The identification of homologs of the majority of the protein complement associated with the membrane trafficking system as well as some jortnalogs in the proteomes of the new *Mantamonas* species (details in Methods, Analysis of the conservation of the membrane-trafficking system complement) corroborated the high completeness of our genomic and transcriptomic datasets, and suggests that these datasets may provide interesting insights in the evolution of anciently originated protein machineries. Overall, our new *Mantamonas* nuclear genome and transcriptome sequences provide high quality data for a major, yet poorly known, eukaryotic supergroup. They will allow more comprehensive comparative studies of genetic diversity in microbial eukaryotes and a better understanding of deep eukaryotic evolution.

## Methods

### Isolation and microscopy of *Mantamonas sphyraenae* sp. nov

*Mantamonas sphyraenae* SRT-306 was collected on 26 Sep. 2013 from the surface of a barracuda caught in a lagoon on Iriomote Island, Taketomi, Okinawa Prefecture, Japan (24° 23′ 36.762″ N, 123° 45′ 22.572″ E). It was isolated manually from the rough sample with a micropipette, and maintained in Erd-Schreiber medium^11^ fortified with 2.5% (final volume) freshwater Cerophyl medium (ATCC 802). Stock cultures were kept in 8 ml volumes in 25 ml culture flasks at 16–18 °C, and transferred at three-week intervals. Bulk cultures were grown at room temperature in 10 cm Petri plates containing ~10 ml medium.

Live cells were observed on an Zeiss Axiovert 100 M inverted microscope equipped with DIC and phase contrast optics. Images were captured with an Olympus DP73 17.28-megapixel camera. Morphometric data were obtained at 1,000x magnification on 20 cells.

### Morphological description of *Mantamonas sphyraenae* sp. nov

*Mantamonas sphyraenae* cells exhibited three general morphologies: ‘balloons’, which were typically ~5 µm long and ~3 µm wide, with a circularly curved anterior and a posterior end tapering to a point; ‘kites’, which were roughly diamond-shaped, about 3.5–4 µm long and wide; and ‘mantas’, which were 4–5 µm wide and ~3 µm long, having a broadly curved anterior end, a more tightly rounded right side, a bluntly rounded projection on the left side, and a posterior comprising either straight edges culminating in a point, two shallowly concave curves, or one of each. All three morphologies were plastic to some extent, although ‘mantas’ were noteworthy in that the left-side projection appeared rigid, and the curved right side frequently very plastic. Intermediates between the three morphologies were sometimes observed. In general, all cells in any given culture flask exhibited the same morphology, which often changed from one observation to the next, one to three weeks later. Exceptions to the prevalent morphology were almost always intermediate forms. We did not observe active transitions from one cell type to another, including to or from intermediate forms. Cells of all morphologies glided slowly and with constant speed, although occasionally stopping; the cell body frequently deformed when changing direction or colliding with other objects.

In all cases, a flagellum, 6–10 µm long, trailed behind the cell, always in a straight line except when the cell was turning, in which case it followed the cell’s path. No movement of the flagellum was seen besides this. Under extremely favourable conditions, a second flagellum could be seen projecting from the anterior-left of ‘manta’ cells, at about a 45° angle. This second flagellum was invariably very thin, stiff, and 1–2 µm long. Very occasionally, we observed an additional protrusion, about the full width of a flagellum and about 1–2 µm long. This was always seen projecting from the posterior of the cell, immediately to the left of, and usually parallel to, the posterior flagellum. It appeared entirely static, and never appeared to change its length or orientation. Cysts were never observed at any stage of culture. Likewise, we never observed dividing cells.

### *Mantamonas sphyraenae* nucleic acid extraction and genome/transcriptome sequencing

To obtain nucleic acids, initially, five plates were inoculated with 500 µl from mature stock cultures. When these had reached high density (qualitatively determined), for each plate, the supernatant was discarded, cells were collected with the use of disposable cell scrapers, and the resulting 0.3–0.5 ml of concentrated cells were inoculated into 50 ml of fresh medium, which was then distributed into five new plates. This process was repeated, for a final count of 125 plates, for DNA extraction and 14 plates used for RNA extraction. For both preparations, cells were harvested with disposable cell scrapers and resuspended in sterile medium. The resuspension was prefiltered using 5.0-µm-pore polycarbonate filters, to remove bacterial flocs, and refiltered using 0.8-µm-pore filters, to remove individual bacteria.

For DNA extraction, filters were incubated in lysis buffer (50 mM Tris, 5 mM EDTA, 50 mM NaCl, pH 8), proteinase K (~300 µg/ml final concentration) and SDS (1% final concentration) for 1 hr on a rotator at 37°. The resulting solution was divided into two aliquots. From these, DNA was extracted in parallel using phenol/chloroform/isoamyl alcohol (25:24:1), extracted again using chloroform/isoamyl alcohol (24:1) and precipitated overnight in 95% EtOH at −20. The DNA was then pelletted in a centrifuge at 4°, washed with 80% EtOH, and resuspended in ddH_2_O. The total yield was ~90 µg.

Long-read genomic sequences were obtained by using Single Molecule Real Time (SMRT) cell technology in a PacBio RSII system at the Cold Spring Harbor Laboratory. A total of 2,304,908 reads (18.7 Gbp) were acquired from 33 SMRT cells. Additional DNA samples were used to prepare two Illumina Nextera short-insert and mate-pair libraries following the manufacturer’s protocols. The sequencing was done with the HiSeq 2500 System and a PE150 run option. A total of 62,929,978 read pairs (18.9 Gbp) and 53,901,870 read pairs (16.2 Gbp) were generated for the paired-end library and the mate pair library, respectively. For RNA extraction, the cell filters were incubated in TRI Reagent (Sigma-Aldrich) and RNA was isolated according to the manufacturer’s instructions, using spin columns for elution. The total RNA sample was subjected to poly-A selection followed by Illumina TruSeq RNA library preparation and a total of 24,187,884 read pairs (7.3 Gbp) were sequenced using the Illumina HiSeq 2500 platform and a PE150 run option. All the genomic and transcriptomic Nextera library preparation and sequencing were conducted at the Weill Cornell’s Genome Resources Core Facility.

### *Mantamonas sphyraenae* genome assembly, gene prediction and ploidy analysis

As the presence of co-cultured bacterial contamination in the sequencing data was expected, b oth the PacBio and Illumina reads were screened for contamination (see details in the technical validation section) and more than 60% of the original data identified as contaminant was discarded (see technical validation section). After this initial decontamination step, a total of 5.89 Gbp of long- read data was assembled using the Canu^5,12^ and FALCON^6^ pipelines.

The resulting genomic contigs from the Canu and FALCON approaches were then polished by aligning the screened PacBio reads to the draft genome using minimap2^13^ and generating a consensus with Racon v1.3.1^14^. Subsequently, a second step of polishing was performed with the high quality Illumina reads by mapping them with bwa-0.7.15^15^ and using Pilon v1.22^16^ to correct for single base errors.

Additionally, MaSuRCA v3.2.6^7^ was used to generate a hybrid assembly using the PacBio as well as the short-insert and mate-pair Illumina data that were retained after bacterial read filtering.

After these assembly efforts, any remaining bacterial contigs were identified by using a combination of homology searches and tetramer frequency-based binning (see details in the technical validation section). From the Canu assembly, a contig corresponding to mtDNA was identified and removed. Clean assemblies were then assessed based on their contiguity and completeness (Table 2) and the FALCON assembly was chosen for further analyses. Because of the specific parameter set utilized, our FALCON analysis did not assemble mtDNA due to its much higher sequence coverage compared to that for the nuclear DNA.

A custom library of repetitive elements was generated for the polished and cleaned nuclear genomic sequence by combining the results of RepeatModeler2^17^ and Transposon-PSI (http://transposonpsi.sourceforge.net/) pipelines. The gathered repeat sequences from both analyse s were merged and clustered to generate a single consensus and refined repeat library that was further compared against the Dfam database^18^ to classify the repetitive elements using RepeatModeler^17^ refiner and classifier modules. Repetitive elements identified by this procedure were then masked out of the nuclear genome using RepeatMasker^17^ before the prediction of protein-coding genes. Subsequently, the RNA-seq libraries were mapped against the genome sequence with HISAT-2^19^ to generate spliced alignments, and BRAKER2^20^ was employed to predict the nuclear protein coding genes integrating the extrinsic evidence from the RNA-Seq data.

Ploidy was inferred by assessing the distribution of allele frequencies at biallelic single nucleotide polymorphisms (SNPs) visually, and with modeling^21,22^ using nQuire^22^. Briefly, the Nextera Illumina reads were mapped to the final genome assembly with Bowtie2 v2.3.5.1^23^ and the resulting.bam file was used to calculate base frequencies for each biallelic site. These results were denoised using nQuire. The resulting frequencies were plotted in R version 3.3.3^24^. Finally, we ran the nQuire’s Gaussian Mixture Model (GMM) command, which models the distribution of base frequencies at biallelic sites, and uses maximum likelihood to select the most plausible ploidy model (Table 3).

### Isolation and microscopy of *Mantamonas vickermani* sp. nov

*Mantamonas vickermani* CRO19MAN was isolated from a sediment sample collected in July 2014 from the shallow marine lagoon Malo jezero (42°47′05.9“N 17°21′01.3“E) on the island of Mljet (Croatia, Mediterranean Sea). The sample was taken from the upper layer of the sediments at the shore of the lagoon with a sterile 15 ml Falcon tube at a depth of 10 cm below the water surface and stored at −20 °C. In September 2019, a small amount of sediment was inoculated in a Petri dish with 5 ml of sterile seawater supplemented with 1% YT medium (100 mg yeast extract and 200 mg tryptone in 100 ml distilled water, as in the protocol from the National Institute for Environmental Studies [NIES], Japan). After observation of some mantamonad cells, serial dilution was performed in a multiwell culture plate to further enrich the culture. We transferred 250 µl of culture to a well with 1 ml of fresh 1% YT seawater medium and then retransferred the same volume to a new well, repeating the process 5 times for a total of 24 wells. Single mantamonad cells were then isolated from one of the enriched cultures with an Eppendorf PatchManNP2 micromanipulator using a 65 µm VacuTip microcapillary (Eppendorf) and a Leica Dlll3000 B inverted microscope. This cell was inoculated into 1 ml of growth medium and after 48 hr incubation we confirmed an established monoculture of *M. vickermani* CRO19MAN.

Optical microscopy observations were performed with a Zeiss Axioplan 2 microscope equipped with oil-immersion differential interference contrast (DIC) and phase contrast objectives. Images were acquired with an AxiocamMR camera using the Zeiss AxioVision 4.8.2 SP1 suite. Videos were recorded using a Sony α9 digital camera. Morphometric data were obtained at 1,000x final magnification on 20 cells. Images were captured at multiple focal planes in order to visualise different cell parts. Measurements of flagella pertain to the visible parts, i.e., the posterior flagellar length is measured beginning from the point at which it emerges from underneath the cell at the body’s posterior end.

### Morphological description of *Mantamonas vickermani* sp. nov

*Mantamonas vickermani* cells are ∼3 μm wide and ∼3.5 μm long; thus noticeably smaller than those of *Mantamonas plastica* (∼5 μm wide and ∼5 μm long) (Glücksman *et al*.^1^) (Fig. 1g–l). Like *M. plastica, M. vickermani* also has a strongly flattened and plastic morphology. However, the characteristic blunt projection on the left-hand side of the cell observed in *M. plastica* is less conspicuous in *M. vickermani*, and not always observed in cells possessing an overall spherical to oval morphology (Fig. 1). The anterior flagellum of *M. vickermani* is ∼2 μm long, rigid in all of its length, and vibrates with a small amplitude; its posterior flagellum is ∼7 μm long and considerably thicker than the anterior one, having a very small acroneme that when seen is never longer than 1–2 μm. Both flagella are also shorter than those reported for *M. plastica* (∼3 μm anterior and ∼10 μm posterior).

*Mantamonas vickermani* glides in a smooth and continuous manner on the substrate with a similar speed and turning behavior to that observed for *M. plastica* (Glücksman *et al*.^1^; AAH, pers. obs.) (Movie 1 and Movie 2). As with *M. plastica, M. vickermani* is a bacterivore with a voracious appetite, engulfing bacteria at a high rate. Interestingly, and in contrast with Glücksman *et al*.^1^, we did observe one cell possessing two posterior flagella, which strongly suggests that it was undergoing cellular division (Fig. 1).

### *Mantamonas vickermani* RNA purification and transcriptome sequencing

This new strain was grown for a week in 75 cm^2^ cell culture flasks with ~10 ml of medium. Fully grown cultures were collected by gently scratching the bottom of the flasks with a cell scraper to resuspend the gliding flagellates and pooled in 50 ml Falcon tubes to be centrifuged at 10 °C for 15 minutes at 15,000 g. Total RNA was extracted from cell pellets with the RNeasy mini Kit (Qiagen) following the manufacturer protocol. Two cDNA Illumina libraries were constructed after polyA mRNA selection, and these were sequenced using the paired-end (2 × 125 bp) method with Illumina HiSeq 2500 Chemistry v4 (Eurofins Genomics, Germany).

### Transcriptomes assembly and proteome prediction

The transcriptomic sequence of *M vickermani* and *M. sphyraenae* were assembled *de novo* using Spades v3.13.1^25^ with the *rna* mode and default parameters specified. Transcripts were then screened to identify remaining contaminants using the Blobtools2^26^ pipeline and homology searches against a custom database (see technical validation section). Predicted proteins were obtained from the clean transcripts using Transdecoder v2 (http:transdecoder.github.io) alowing for a single prediction by transcript (–single-bes-only option) and using the universat genetic code. Subsequently, CD-HIT^27^ clustering was employed (with a threshold of > = 90% of identity) to produce a non-redundant data set of proteins for each of the trancriptomes, and to eliminate falsely duplicated proteins stemming from alternatively spliced transcripts.

### Phylogenomic analyses

The dataset of 351 conserved protein markers from Lax *et al*.^3^ was updated by BLASTP searches^28^ against the inferred proteomes of representatives of other eukaryotic lineages, including the proteomic data for our two new mantamonad strains. Each protein marker was aligned with MAFFT v.7^29^ and trimmed using TrimAl^30^ with the -automated1 option. Alignments were manually inspected and edited with AliView^31^ and Geneious v6.06^32^. Single-protein trees were reconstructed with IQ-TREE v1.6.11^33^ under the corresponding best-fitting model as defined by ModelFinder^34^ implemented in IQ-TREE^33^. Each single-protein tree was manually inspected to discard contaminants and possible cases of horizontal gene transfer or hidden paralogy. At the end of this curation process, we kept a final taxon sampling of 14 species, including members of Ancyromonadida, Malawimonadida, Opisthokonta, and CRuMs (concatenated alignment and supplementary trees are available at Figshare^35^), and 182 protein markers that were present in all mantamonad species (with at least 80% of markers identified in each taxon). All proteins were realigned, trimmed as previously described, and concatenated, creating a final supermatrix with 62,088 amino acids.

A Bayesian inference tree was reconstructed using PhyloBayes-MPI v1.5a^36^ under the CAT-GTR model^37^, with two MCMC chains, and run for 10,000 generations, saving one of every 10 trees. Analyses were stopped once convergence thresholds were reached (i.e. maximum discrepancy <0.1 and minimum effective size >100, calculated using bpcomp). Consensus trees were constructed after a burn-in of 25%. Maximum likelihood (ML) analyses were done with IQ-TREE v1.6.11^33^, first by calculating the ML tree under the LG+F+R4 model, which was used as guide tree for the PMSF approximation^38^ run under the LG+C60+F+R4 model.

**Fig. 4:**
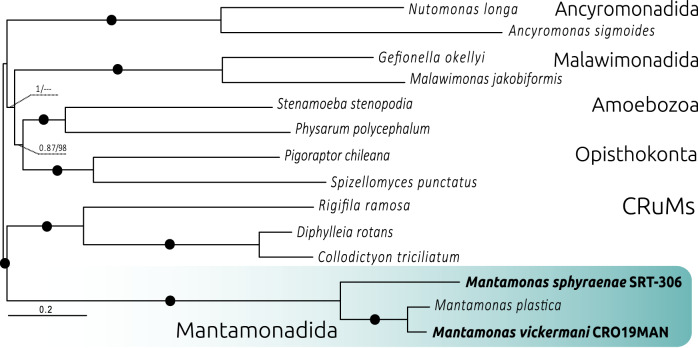
Phylogenomic analysis of CRuMs clade. Bayesian inference (BI) phylogeny based on 182 conserved proteins from Lax *et al*.^3^. The tree was obtained using 62,088 amino acid positions with the CAT-GTR model. Statistical support at branches was also estimated using maximum likelihood (ML) under the LG+C60+F+R4 model with the PMSF approximation. Numbers at branches indicate BI posterior probabilities and ML bootstrap values, respectively; bootstrap values <50% are indicated by dashes. Branches with support values higher than or equal to 0.99 BI posterior probability and 95% ML bootstrap value are indicated by black dots. The tree was rooted between CRuMs and everything else.

Consistent with previous studies, our maximum likelihood (ML) and Bayesian inference (BI) phylogenetic trees recovered the monophyly of CRuMs with high BI posterior probability (0.99) and ML bootstrap support (95%), although it is worth noticing that the outgroup is highly reduced since resolving the position of CRuMs in the tree of eukaryotes is outside the scope of this paper. The monophyly of *Mantamonas* received full support from both methods. We found Mantamonas sphyraenae to be sister to a maximally-supported clade containing *M. plastica* and *M. vickermani* (Fig. 4).

### CRuMs orthologue analysis and protein functional annotation

**Fig. 5:**
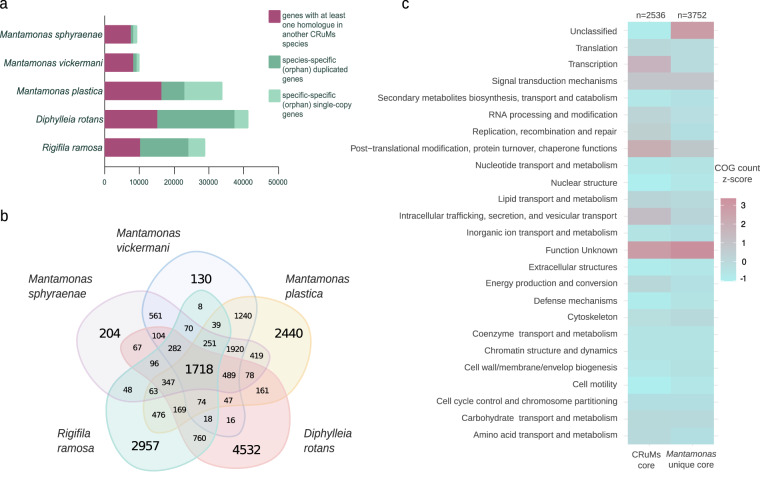
Orthology analysis across the CRuMs supergroup. (**a**) Distribution of coding sequences shared among CRuMs representatives (magenta) or that are species-specific in one or several copies (dark and light green, respectively). Note that these numbers do not represent genes but open reading frames identified in assembled transcripts, except for *M*. *sphyraenae*. (**b**) Number of orthogroups shared among compared CRuMs species. (**c**) COG functional categories associated with orthogroups shared among all CRuMs, and those associated with orthogroups shared across *Mantamonas* species but absent in other CRuMs taxa. COG counts were scaled by column using z-score standardization.

Orthologous gene families were identified among the predicted proteomes of *Mantamonas sphyraenae*, *Mantamonas vickermani* and the publicly available proteomes of *Mantamonas plastica, Diphylleia rotans* and *Rigifila ramosa* as obtained from the EukProt v3 database^39^ using OrthoFinder v2.5.4^40^. For this, we used DIAMOND^41^ (“ultra-sensitive” mode, and query cover > = 50%), an inflation value of 1.5, and the MCL clustering algorithm (Fig. 5a).

Then, the predicted proteomes of *M. sphyraenae, M. vickermanii*, *M. plastica*, *D. rotans*, and *R. ramosa* were functionally annotated with the EggNOG-mapper pipeline^9^, using DIAMOND ultra-sensitive mode and all domains of life as the target space. During this process, individual sequences composing the CRuMs orthogroups generated by OrthoFinder were assigned a COG functional category. This information was summarized at the orthogroup level by assigning to each orthogroup a single COG category corresponding to the most frequent annotation of its individual sequences, provided that it represented at least 50% of the sequences within the orthogroup.

A total of 1,718 orthogroups were found to be conserved among all CRuMs taxa (Fig. 5b), while 4,378 were identified as shared between the three *Mantamonas* species, representing the minimal core proteome of the genus *Mantamonas* as currently known, among which 2,161 orthogroups are not found in the other two CRuMs lineages. Our species also display a smaller number of unique proteins than the publically available proteomes likely due to the methodological strategy that we employed to assemble the transcriptomes and infer open reading frames that reduces the number of short and incomplete ORFs and sioforms when compared with the proteomes derived from the other CRuMs transcriptomes. However, beyond the absolute numbers of predicted coding sequences, the comparison between all these proteomes gives us an indication about the degree of the diversity of gene content in each of our two *Mantamonas* species.

Most of the proteins conserved among the CRuMs taxa (99.6%) were found to have an ortholog in the EggNOG database and to belong to at least one Cluster of Orthologous Groups (COG)^42,43^ functional category, where the most highly represented were “Function unknown” and “Post-translational modificationand “Intracellular trafficking” (Fig. 5c). By contrast, a substantial amount of orthogroups conserved among mantamonads (12%), but absent in other CRuMs lineages, could not be assigned to any cluster in the EggNOG database. In addition, most orthogroups conserved in mantamonads but absent in other CRuMs that could be connected to an existing EggNOG cluster were annotated as “Function unknown” (Fig. 5c). Altogether, this large number of *Mantamonas*-specific genes of unknown function suggests that many genetic innovations occurred at the origin of this group.

### Analysis of the conservation of the membrane-trafficking system complement

To assess the complement of the membrane trafficking system encoded in our *Mantamonas* genome and transcriptome datasets, we performed homologous searches of a selection of protein query sequences from the genomes of *Homo sapiens* (GCF_000001405.40), *Dictyostelium discoideum* (GCF_000004695.1), *Arabidopsis thaliana* (GCF_000001735.4) and *Trypanosoma brucei* (GCF_000002445.2) available at the GenBank of the NCBI (National Center for Biotechnology Information) database. These proteins included components of the machinery for vesicle formation (HTAC-derived coats, ESCRTs, and ArfGAPs) and vesicle fusion (SNAREs and SM proteins, TBC-Rab GAPs, and Multi-subunit tethering complexes)^10^.

BLASTP and TBLASTN were used to search the predicted proteomes and nucleotide coding sequences, respectively, of *M. sphyraenae* and *M. vickermani*. The HMMER3 package was used to find more divergent protein sequences using the hmmsearch tool^44^. In cases in which only TBLASTN hits were retrieved, these were translated using Exonerate^45^. Potential orthologs (i.e., hits with an E-value below 0.05) were further analyzed by the Reciprocal Best Hit (RBH) approach, using the *Mantamonas* candidate orthologs as queries against the *H. sapiens*, *D. discoideum* and *A. thaliana* proteomes. If the best hit was the protein of interest and had an E-value two orders of magnitude lower than the next non-orthologous hit, this was considered as orthology validation. Forward and reverse searches were performed using the AMOEBAE tool^46^.

**Fig. 6:**
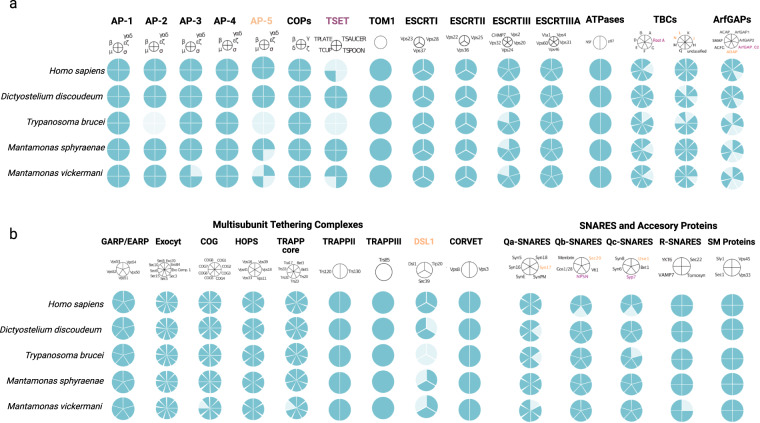
Distribution of proteins associated with the membrane trafficking system in new *Mantamonas* species and other model organisms. (**a**) Selected vesicle formation machinery; (**b**) Selected vesicle fusion machinery. Names of proteins with jotnarlogs are in purple; those with patchy distribution are in orange.

We detected most proteins of the membrane-trafficking system in the two new *Mantamonas* species, making it one of the most complete known protein complements for this system. Notably, when compared to representatives of well-characterized model organisms from other supergroups (Fig. 6). *Mantamonas* encodes some rarely retained proteins, such as the AP5 complex^47^ and syntaxin 17^48^. We also identified several jotnarlogs (Fig. 6), including a near-complete TSET complex, and the SNAREs NPSN and Syp7^35^.

## Data Records

The read data associated with the nuclear genome and transcriptomic datasets of *Mantamonas sphyraenae* and the transcriptome of *Mantamonas vickermani* have been submitted to the NCBI SRA database^49^ (Table 4).

**Table 4:** Summary of sequencing data records.

Species and strain name	Type	Platform	Read type	SRA accession number
*M. sphyraenae* STR306	DNA	PacBio RS II	Single molecule	SRR21818797
*M. sphyraenae* STR306	DNA	Illumina HiSeq 2500	Paired	SRR21818798
*M. sphyraenae* STR306	DNA	Illumina HiSeq 2500	Mate pair	SRR22188164
*M. sphyraenae* STR306	RNA	Illumina HiSeq 2500	Paired	SRR21818794
*M. vickermani* CRO19MAN	RNA	Illumina HiSeq 2500	Paired	SRR21818793

The Transcriptome Shotgun Assemblies have been deposited at DDBJ/EMBL/GenBank under the accessions GKLA00000000 and GKKZ00000000 for *M. vickermani* and *M. sphyraenae* respectively. The final nuclear genome assembly of *Mantamonas sphyraenae* has been deposited at GenBank under the accession GCA_026936335.1^50^. The versions described in this paper are the first versions. The prediction of protein-coding genes from the genome and transcriptome assemblies of *Mantamonas sphyraenae*, as well as from the transcriptome assembly of *M. vickermani* are available at Figshare^35^.

Phylogenomic analysis alignments and trees, and membrane-trafficking predicted proteins table can be found on Figshare^35^.

## Technical Validation

### Quality assessment of sequencing datasets

All Illumina paired-end raw reads used for genome polishing were quality-checked with FastQC v0.11.8^51^ and trimmed using TRIMMOMATIC^52^ to retain only reads with maximum quality scores. PacBio reads resulted in an N50 of 11,048 bp and an average coverage of 106x after filtering out the identified contaminant sequences (see below).

### Identification and filtering of contaminant sequences

Mantamonads grow in non-axenic cultures with co-cultured prokaryotes on which they feed. Therefore, various methods were employed to ensure the correct identification and filtering of contaminant sequences in the genomic and transcriptomic datasets of *M. sphyraenae* and *M. vickermani*.

For the genomic dataset of *M. sphyraenae*, we first identified the main bacterial contaminants from the initial genome assemblies^53^. In addition, we established a custom database consisting of contigs assembled from Illumina sequencing data from bacteria only enrichment cultures derived from the lab’s several xenic protist cultures. These were used to screen PacBio reads using BLASR v5.1^54^ The Illumina reads were screened similarly using Bowtie2 v2.3.5.1^23^. Only Illumina reads in which neither pair aligned to the bacterial database were retained for further assembly recovering 57% and 49% of the pair-end and mate pair reads from the total libraries respectively.

After genome assembly using the filtered reads, remaining contaminant contigs were identified by using MyCC v1^55^, which bins contigs based on their tetranucleotide frequencies and coverage. Clusters were formed using the affinity propagation (AP) algorithm and visualized in a 2-dimensional Barnes-Hut-SNE plot. BLASTN searches using default parameters were conducted against the ‘nt’ database from the NCBI to taxonomically classify the bins. Contigs were identified as contaminants if they contained no hits other than to prokaryotes, and if they were clustered away from the main eukaryotic bin. Finally mitochondrial sequences were screened out from the short and long read libraries of *M. sphyraenae* by mapping them against the mitochondrial genome using bwa-0.7.15^15^ and minimap2^13^ respectively.

**Fig. 7:**
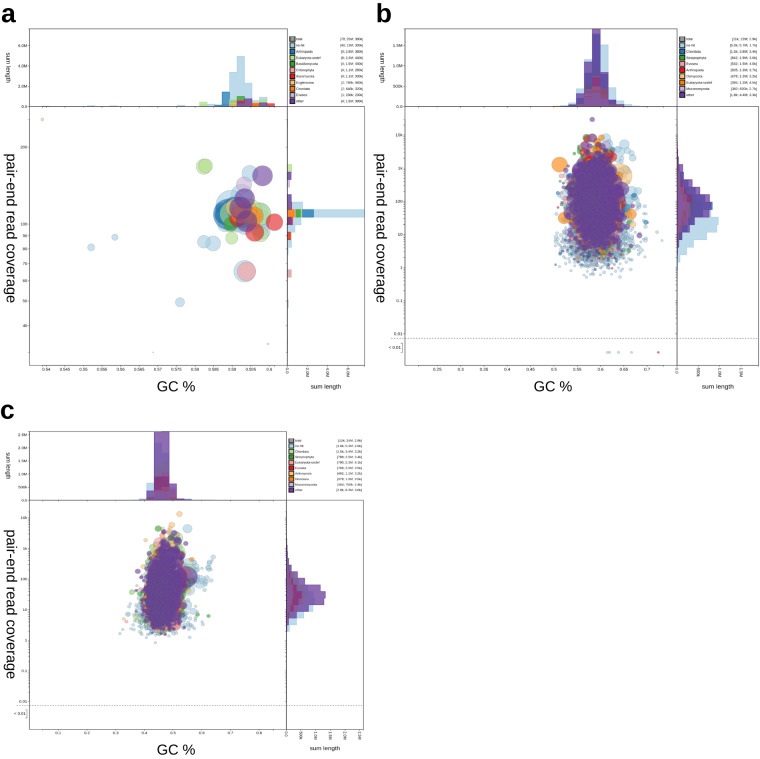
Blob plot of read coverage against GC proportion in genome and transcriptomic contigs. (**a**) *M. sphyraenae* genomic sequences. (**b**) *M. sphyraenae* transcripts. (**c**) *M. vickermani* transcripts. Records are coloured according to their similarity to different phyla. Circles are sized in proportion to records cumulative length. The assembly has been filtered to exclude records whose taxonomic assignment matches “Bacteria”. Histograms show the distribution of record length sums along each axis.

The assembled transcriptomes of *M. sphyraenae and M. vickermani were* decontaminated with the Blobtools2 pipeline^26^. Briefly, this approach helps to identify contaminant sequences based on their biases in coverage and GC content, as well as on a taxonomic classification established by DIAMOND searches^41^ against the ‘nt’ and Uniprot databases^56^. In addition, a second cleaning step was done by performing DIAMOND searches against a database containing all the proteins of the prokaryotic Genome Taxonomy Database (GTDB)^57^ and the eukaryotic-representative EukProt v3 database^39^. A protein was considered as a probable contaminant and excluded from further analyses if its best hit corresponded to any protein from GTDB, with strict cutoffs of identity ≥50% and query coverage ≥50%. Finally, a blobplot was generated for the final genomic and transcriptomic contigs of *M. sphyraenae* and *M. vickermani*, respectively, to verify the absence of contaminant sequences (Fig. 7).

### Completeness analysis

To assess the completeness of the decontaminated genome and transcriptome datasets, we employed the BUSCO v5.3.2 pipeline. We identified the percentage of near-universal single copy orthologs of the eukaryote_odb10 database^12^ on the predicted proteomes of *M. sphyraenae* and *M. vickermani*, as well as those of other species belonging to the CRuMs supergroup available in the EukProt v3^39^ database for comparison purposes (Fig. 2). Moreover, the comparison of the transcriptomic dataset and the genomic dataset of Mantamonas sphyraenae revealed that 96% of the proteins predicted in the transcriptome share similarity with the proteins derived from the genome (80% of these being identical) and 271 proteins were found to be present uniquely in the transcriptome. Adittionally, the mapping coverage from the clean transcriptomic reads to the genome sequence was of 97.38%, suggesting a near complete representation of the gene space in the genome-predicted proteins.

### Data usage notes

Formal species descriptions

All taxonomic descriptions in this work were approved by all authors.

Eukarya: ‘CRuMs’

Order Mantamonadida Cavalier-Smith 2011

Family Mantamonadidae Cavalier-Smith 2011

Genus *Mantamonas* Cavalier-Smith and Glücksman 2011

#### *Mantamonas sphyraenae* sp. nov

Description: Cells with varying morphologies: shaped as manta rays (as for genus in Glücksman *et al*.^1^), ~3 µm long and ~5 µm wide; diamonds, 4 ± 1 µm in both dimensions; or rounded anteriorly and tapering posteriorly, ~5 µm long and ~3 µm wide. Anterior flagellum stiff, 0.5–1.0 µm long. Other characters as for genus.

Type culture: SRT306

Type locality: Surface of barracuda caught in lagoon on Iriomote Island, Taketomi, Okinawa Prefecture, Japan (24° 23’ 36.762″ N, 123° 45’ 22.572″ E).

Isolator: Takashi Shiratori

Etymology: From Sphyraena, the genus name for barracuda, the fish from which the type strain was obtained.

Gene sequence: The nuclear genome and transcriptomic read sequencing data from *Mantamonas sphyraenae* (strain SRT306) were deposited in GenBank under BioProject accession number PRJNA886733.

#### *Mantamonas vickermani* sp. nov

Description: Cell size ∼3 μm (2.5–4.3 μm) long, ∼3.5 μm (3.0–4.0 μm) wide; cells almost perfectly round, although in some cases possessing a small projection to the left side of the cell; without pseudopodia; anterior flagellum usually ≤2 μm long (1.2–2.7 μm), held forwards and to left ∼40–50° to longitudinal axis, does not beat except for slight terminal vibration; posterior flagellum ∼7 μm long (6–8.9 μm), conspicuous and sometimes acronematic. Other characters as for genus.

Type culture: CRO19MAN

Type locality: Specimen isolated from the sediments of the marine lake Malo jezero in the island of Mljet, Croatia.

Isolator: Luis Javier Galindo.

Etymology: The name vickermani honors work on heterotrophic protists by Keith Vickerman.

Gene sequence. The full transcriptome read data from *Mantamonas vickermani* (strain CRO19MAN) were deposited in GenBank under BioProject accession number PRJNA886733.

## Data Availability

All the employed software as well as their versions and parameters were described in the method section. If no parameters were specified, default settings were employed. Data visualization plots were generated using R v4.1.2 (https://cran.r-project.org/, R development core team) and https://bioinformatics.psb.ugent.be/webtools/Venn/.
